# Stable reconstructed human gingiva–microbe interaction model: Differential response to commensals and pathogens

**DOI:** 10.3389/fcimb.2022.991128

**Published:** 2022-10-20

**Authors:** Yan Zhang, Lin Shang, Sanne Roffel, Bastiaan P. Krom, Susan Gibbs, Dongmei Deng

**Affiliations:** ^1^ Department of Oral Cell Biology, Academic Centre for Dentistry Amsterdam (ACTA), University of Amsterdam and Vrije Universiteit Amsterdam, Amsterdam, Netherlands; ^2^ Department of Preventive Dentistry, Academic Centre for Dentistry Amsterdam (ACTA), University of Amsterdam and Vrije Universiteit Amsterdam, Amsterdam, Netherlands; ^3^ Department of Orthodontic, Affiliated Stomatology Hospital of Guangzhou Medical University, Guangzhou Key Laboratory of Basic and Applied Research of Oral Regenerative Medicine, Guangzhou, China; ^4^ Department of Molecular Cell Biology and Immunology, Amsterdam Infection and Immunity Institute, Amsterdam University Medical Centre, Vrije Universiteit Amsterdam, Amsterdam, Netherlands

**Keywords:** *Streptococcus gordonii*, *Aggregatibacter actinomycetemcomitans*, tissue engineering, gingiva, organotypic, host-microbe

## Abstract

**Background:**

To investigate human oral health and disease, models are required which represent the interactions between the oral mucosa and microbiome. Our aim was to develop an organotypic model which maintains viability of both host and microbes for an extended period of time.

**Methods:**

Reconstructed Human Gingiva (RHG) were cultured air-lifted with or without penicillin-streptomycin (PS) and topically exposed to *Streptococcus gordonii* (commensal) or *Aggregatibacter actinomycetemcomitans* (pathogen) for 72 hours in agar. RHG histology, viability and cytokines (ELISA), and bacterial viability (colony forming units) and location (FISH) were assessed.

**Results:**

The low concentration of topically applied agar did not influence RHG viability. Topically applied bacteria in agar remained localized and viable for 72 hours and did not spill over to infect RHG culture medium. PS in RHG culture medium killed topically applied bacteria. Co-culture with living bacteria did not influence RHG viability (Ki67 expression, MTT assay) or histology (epithelium differentiation, Keratin10 expression). RHG exposed to *S. gordonii (*with or without PS) did not influence low level of IL-6, IL-8, CCL2, CCL5, CCL20 or CXCL1 secretion. However, all cytokines increased (except CCL2) when RHG were co-cultured with *A. actinomycetemcomitans*. The effect was significantly more in the presence of living, rather than dead, *A. actinomycetemcomitans*. Both bacteria resulted in increased expression of RHG antimicrobial peptides (AMPs) Elafin and HBD-2, with *S. gordonii* exposure resulting in the most Elafin secretion.

**Conclusion:**

This technical advance enables living human oral host–microbe interactions to be investigated during a 72-hour period and shows differences in innate immunology triggered by *S. gordonii* and *A. actinomycetemcomitans*.

## Introduction

Humans are host to a complex flora of microorganisms that colonize the oral mucosa and gingiva. More than 700 species of commensal and pathogenic, aerobic and anaerobic bacteria inhabit the oral cavity (Aas et al., 2005). The healthy oral cavity is a result of a delicate balance between the host and these microbes.

*Streptococcus gordonii* is a Gram-positive facultative anaerobic bacterium which has adapted to a commensal role during a long evolutionary relationship with the host. *S. gordonii* is one of the initial colonizers of dental plaque, and is also one of the streptococcal groups which dominates the oral mucosa of healthy individuals (Díaz et al., 2012). However, a subset of *S. gordonii* can escape the host restraint mechanisms and initiate oral infectious diseases. For example, by adhering to *Candida albicans* hyphae, the two microbes form bacteria-fungal biofilms which are highly antibiotic resistant and invasive (Montelongo-Jauregui et al., 2019; Salvatori et al., 2020). *S. gordonii* is also considered to be a direct precursor of periodontal disease inhabiting the subgingival crevice (Cook et al., 1998; Kuboniwa and Lamont, 2010). For these reasons, *S. gordonii* is referred to as an opportunistic commensal (Boman, 2000; Dixon et al., 2004).

In contrast, *Aggregatibacter actinomycetemcomitans* is strongly associated with the pathogenesis of aggressive periodontitis (Mans et al., 2006; Casarin et al., 2010). It is a Gram-negative facultative anaerobic coccobacillus that exerts specific effects on the host mucosal immune system and is associated with the development of oral infectious diseases (Henderson et al., 2002; Herbert et al., 2016). Numerous virulence factors are produced by *A. actinomycetemcomitans* that affect the gingival epithelium and trigger the onset of periodontitis, including lipopolysaccharide (LPS), leukotoxin, cytolethal-distending toxin, collagenase and outer membrane protein (Gholizadeh et al., 2017). *A. actinomycetemcomitans* is also considered to play an important role in the initiation of biofilm that is potentially pathogenic (Díaz and Kolenbrander, 2009).

When bacteria invade into the oral mucosa barrier, the host innate immunity is rapidly activated as a first line of defense, with gingival epithelial cells playing an important role (Dickinson et al., 2011; Moutsopoulos and Konkel, 2018). Bacteria can bind to host receptors e.g., toll-like receptors (TLRs) on the epithelial cell membrane, leading to a cascade of intracellular pathways which may ultimately result in the release of pro-inflammatory cytokines and chemokines such as interleukin (IL)-6, IL-8, CCL2, CCL5, CCL20 and CXCL1 (Schaller et al., 2002; Teles et al., 2009; Dickinson et al., 2011; Lundmark et al., 2019). The level of these functional molecules, which in turn determines the extent and type of immune reaction has been shown to indicate the severity of mucosa tissue damage (Teles et al., 2009; Diesch et al., 2021). To maintain health, it is critical that the host strikes a complex balance between tolerating (and even benefitting from) resident commensals and suppressing potential pathogens (Lamont et al., 2018; Moutsopoulos and Konkel, 2018). Therefore, studying how the oral barrier responds to different bacterial challenges will contribute greatly to understanding mechanisms behind this balance.

Animal models have been extensively used to study the interaction between host and oral microbes. For example, *A. actinomycetemcomitans* has been repeatedly verified for its pathogenic roles during the onset and development of periodontitis in rodent periodontitis models, such as the rat infection model, lipopolysaccharide injection model, and even in an advanced humanized mouse model (Graves et al., 2012; Buschhart et al., 2020; Rojas et al., 2021; Wang et al., 2021). However, using experimental animals to study human oral host-microbe interactions has limited translational effectiveness due to differences between humans and animals in the mucosal immunity as well as oral microbiota (Elliott David et al., 2005; Hyde et al., 2014). Furthermore, when preparing periodontitis models, imprecise administration of microbes, risk of mechanical trauma, failure to induce periodontitis in spite of repetitive injection can be problematic. Although oral bacteria, like *S. gordonii* and *A. actinomycetemocomitans*, have been broadly investigated for their host influence *in vitro*, most of these studies used 2D keratinocyte cultures to represent the host (Hasegawa et al., 2007; Sliepen et al., 2009; Stathopoulou et al., 2010; Dickinson et al., 2011). Little is known about how these bacteria affect the host in a human-representative context. Therefore, there is an urgent need to develop human physiologically relevant *in vitro* models to investigate host-microbe interactions.

In the past, we have developed a reconstructed human gingiva (RHG) consisting of a stratified and differentiated gingival epithelium on a fibroblast-populated collagen hydrogel which functions as the lamina propria (Kosten et al., 2015). RHG is cultured exposed to the air from above with culture medium being supplied from below, which enables topical application of chemicals and bacteria similar to the *in vivo* situation (Shang et al., 2020). Using this RHG model, we have investigated host TLR expression and cytokine secretion in response to saliva-derived multi-species biofilms (Buskermolen et al., 2018; Shang et al., 2019), as well as to an oral resident bacterium *Streptococcus mitis* in combination with metal sensitizers related to oral allergy (Shang et al., 2020). Even though this co-culture model represents complex host-microbe interactions in comparison to conventional 2D monolayer models, it still has the major limitation in that bacterial cells were no longer viable after 24 hours. This reduction in viability was most likely caused by the addition of antibiotics (e.g., penicillin-streptomycin (PS)) to the culture medium (Waksman, 1953; Georgopapadakou and Liu, 1980). Antibiotics were added to the culture medium to reduce the risk of bacterial infection caused by bacteria spilling over from the surface of RHG *via* the sides of the transwell into the culture medium below. Taken together, a new strategy which can maintain the viability of topically applied bacterial cells without increasing the risk of extensive bacterial infection is needed to study the host-microbe interactions for an extended period of time.

The first aim of this study was to develop a model to investigate interactions between the RHG and living oral bacteria over an extended period of time of 3 days. The impact of two essential technical steps on RHG viability were investigated: a) the impact of topically applied agar as a vehicle to keep the bacteria localized on the surface of RHG thus preventing bacteria from spilling over into the culture medium *via* the edges of the transwell, and b) the impact of the presence and absence of PS on the host-microbe co-culture over a period of 3 days. The second aim of this study was to investigate differences in innate cytokine immune responses when RHG is exposed to a commensal and a pathogenic bacterium for 3 days. In this study, *S. gordonii* represented the commensal and *A. actinomycetemcomitans* represented the pathogenic bacteria.

## Materials and methods

### Reconstructed human gingiva (RHG)

Immortalized gingiva keratinocyte (KC-TERT, OKG4/bmi1/TERT, Rheinwald laboratory, Boston, USA) (passage 10 to 20) and fibroblast (Fib-TERT, T0026, ABM, Richmond, BC, Canada) (passage 10 to 20) cell lines were used for constructing RHG. The RHG model, consisting of a fibroblast-populated collagen hydrogel (collagen I derived from rat tail) overlaid with keratinocytes (0.5 x 10^6^ cells) was cultured exactly as previously described (Kosten et al., 2015). In brief, RHG were cultured in six-well transwell inserts (pore size 0.4μm, 24mm, Corning, USA), first submerged in culture medium for 3 days and then lifted to the air-liquid interface for an additional 10 days to induce epithelial differentiation and stratification in culture medium containing DMEM/Ham’s F12 (3/1) (Gibco, Grand Island, USA) supplemented with 1% Fetal Clone III (HyClone, GE Healthcare, Chicago, USA), 1% PS (Gibco, Grand Island, USA), 0.1µM insulin, 1µM hydrocortisone, 1µM isoproterenol, 10µM L-carnitine, 10mM L-serine, 0.4mM ascorbic acid, and 2ng/mL epidermal growth factor. All agents were purchased at Sigma-Aldrich (St. Louis, USA) when not specified. One day prior to bacteria exposure, PS and hydrocortisone were omitted in RHG culture medium for some experimental conditions as indicated below.

### Bacterial strains and growth conditions

*S. gordonii* ATCC35105 was routinely cultured in Brain Heart Infusion (BHI, BD Difco, Strasbourg, France) broth or on BHI containing 1.5% agar plates for determination of viable bacterial cell counts as described below. *A. actinomycetemcomitans* Y4 was routinely cultured in BHI containing 0.02M NaHCO_3_ and 1% glucose or on tryptic soy agar (BD Difco, Strasbourg, France) plates containing 5% sheep blood (BA plate). Both strains were cultured anaerobically (10% CO_2_, 10% H_2_, and 80% N_2_) at 37°C.

### RHG exposure to live and dead bacterial cells

RHG underwent 5 exposure conditions as follows: (1) negative control: unexposed RHG, (2) vehicle control: RHG exposed to the vehicle agar (BD Difco, Strasbourg, France), (3) exposed to live *S. gordonii* of high and low densities, (4) exposed to live *A. actinomycetemcomitans* of high and low densities, (5) exposed to heat-killed *A. actinomycetemcomitans* of high density only. For conditions (1-4), the co-cultures were cultured in the presence or absence of PS in the culture medium. For condition (5) where no live *A. actinomycetemcomitans* were present, co-cultures were grown in the absence of PS.

For conditions (3) and (4) where living bacteria were used, prior to exposure to RHG, the stationary cultures of *S. gordonii* and *A. actinomycetemcomitans* were first diluted to 4 x 10^9^ CFU/mL respectively. The dilutions were then mixed with 0.3% agar containing BHI either at the ratio of 1:1 (high bacterial challenge group) or after 100 times dilution (low bacterial challenge group). A drop of the mixture (50µL) was then pipetted onto the center of RHG. The final bacterial cell counts on RHG were either 1 x 10^6^ CFU (low) or 1 x 10^8^ (high) CFU.

For condition (5) with dead bacteria exposure, the stationary culture of *A. actinomycetemcomitans* was first diluted in PBS to 4 x 10^9^ CFU/mL. This dilution was incubated at 60°C for 1 h before mixing with 0.3% agar containing BHI and at the ratio of 1:1. A drop of the mixture (50µL) was then pipetted on top of RHG. The number of heat-killed *A. actinomycetemcomitans* was equivalent to the bacterial cell counts in the living high bacterial group. Heat-killed *A. actinomycetemcomitans* was plated on BA agar to confirm effectivity of heat-killing.

During exposure, all RHG were cultured under aerobic conditions at 37°C, with 7.5% CO_2_ and 95% humidity representing the environment of the oral cavity. The culture medium of all conditions was refreshed and collected on day 1, day 2 and day 3 (every 24 hours). On day 3, each RHG was divided in half; one half was used to assess viable bacterial counts and the other half was used to assess RHG viability and for (immune-)histochemical analysis.

### Viable bacterial cell counts

To determine the number of living bacteria, RHG was homogenized, collected, diluted and plated for colony-forming-unit (CFU) counting. All procedures were performed in a sterile environment. Cysteine peptone water (CPW, 5g/L of yeast extract, 1g/L of peptone, 8.5g/L of NaCl, 0.5g of L-cysteine hydrochloride, pH 7.2) was used for processing the tissue homogenate as well as for serial dilutions before plating.

In detail, RHG in 1mL CPW was mechanically broken down to a homogenous suspension using a glass tissue homogeniser (Fisherbrand™, Göteborg, Sweden). Another 1 ml CPW was added to wash and collect the residual materials. All tissue homogenates were then filtered through a Falcon™ cell strainer (100μm, Corning, NY, USA) in order to remove tissue fragments, and serially diluted with CPW. The filter-through and the dilutions (100µL) were plated on BHI (*S. gordonii*) or BA agar plates (*A. actinomycetemcomitans*) and incubated anaerobically for 3 days at 37°C before CFU were counted.

### MTT assay

Metabolic activity, which is representative of the RHG viability, was quantified using the Thiazolyl blue tetrazolium bromide (MTT) assay as described previously (Bil et al., 2018). RHG biopsies (3mm diameter) of each experimental condition were incubated for 2 hours in a 2mg/mL MTT (Sigma-Aldrich, USA) solution in PBS at 37°C. Then the biopsies were transferred to 200μL acidified isopropanol solution (isopropanol: 0.04M HCl = 3:1) for 24 hours, followed by colorimetric analysis at 570 nm (Mithras LB 940, Berthold Technologies, Austria) to determine the absorbance of the dissolved formazan. A pilot study was carried out to confirm that the bacterial agar mixture alone did not contribute to the MTT signals on day 3.

### Histology and Immunohistochemistry (IHC)

For each experimental condition, a strip of RHG tissue was fixed in 4% paraformaldehyde and processed for paraffin embedment. Tissue sections (5μm) were stained with hematoxylin and eosin (HE) for histological analysis or used for IHC of Ki67 (Dako, Santa Clara, USA), keratin10 (PROGEN Biotechnik, Heidelberg, Germany), Elafin (Hycult Biotech, Uden, The Netherlands) and HBD-2 (OriGene Technologies, Rockville, USA). The stained slides were visualized using a Nikon Eclipse 80i microscope with 20x/0.5 objective (Nikon, Melville, USA). Ki67 quantification was performed as previously described (Gibbs et al., 2000). The Ki67 index of each sample (expressed as percentage) was determined by counting the number of Ki67 positive cells from 100 cells at 4 random locations in the epithelial basal cell layer. Solid phase sandwich Enzyme-Linked Immunosorbent Assay (ELISA, Human Trappin-2/Elafin DuoSet, R&D System, Minneapolis, USA) was used to quantify Elafin secretion in RHG culture medium.

### Fluorescence *in situ* hybridization

Bacteria in RHG sections (5μm) were detected by Fluorescence *In Situ* Hybridization (FISH) following the manufacturer’s protocol (10-ME-H000, BioVisible, the Netherlands). The FISH probe EUB338 (5′-GCTGCCTCCCGTAGGAGT-3′) was used to visualize bacteria. After FISH staining, the tissue sections were mounted with a mounting medium containing DAPI (Fluoroshield, Abcam, Cambridge, UK). The images were taken under a fluorescence microscope with 40x/1.0 objective (Nikon Eclipse 80i, Melville, USA) at Ex/Em= 490/525nm (Probe) and Ex/Em= 375/460nm (DAPI) with NIS-Elements software (Nikon, Amsterdam, the Netherlands) (Shang et al., 2019)

### ELISA

ELISAs were performed as described by the suppliers and as described previously (Spiekstra et al., 2005). In short, RHG culture medium were used to detect cytokine secretion of IL-6, CCL2, CCL5, CCL20 and CXCL1 (all supplied by R&D systems, Minneapolis, USA) and IL-8 (Sanquin, Amsterdam, the Netherlands).

### Statistics

Statistical analysis was performed with GraphPad Prism, version 9.1 (GraphPad Software, Inc., La Jolla, USA). The statistical methods for analyzing the data were described in detail in the figure legends. All data represent at least three individual experiments, each with an intra-experiment duplicate. Differences were considered significant when *p* < 0.05. Data are represented as mean ± SEM; *p<0.05; **p<0.01; ***p<0.001.

## Results

### Optimization of RHG culture conditions to permit 3-day exposure of viable bacteria

**Figure 1:**
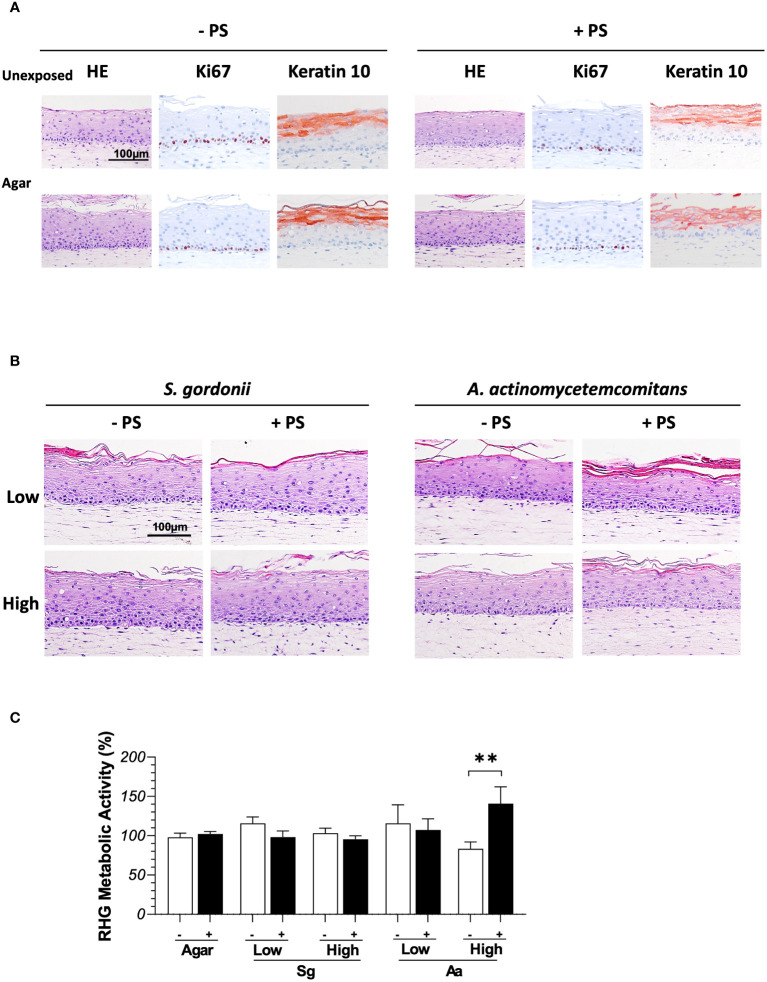
Histology of full thickness RHG after being exposed to bacteria in the presence (+ PS) or absence (- PS) of penicillin and streptomycin in the culture media over 3 days. **(A)** Hematoxylin and eosin staining (HE) and immunohistochemical stainings (Ki67/keratin10) of RHG unexposed or exposed to agar vehicle control. **(B)** HE staining of RHG exposed to *S.gordonii or A.actinomycetemcomitans* at either low or high inoculation density (low: 10^6^ CFU/sample; high: 10^8^ CFU/sample). **(C)** The metabolic activity of RHG exposed to *S. gordonii* and *A. actinomycetemcomitans* are presented relative to agar vehicle control on day 3. Two-way ANOVA followed by Fisher’s LSD test was performed to determine the influences of PS and bacteria density on RHG metabolic activity. **p<0.01. Data represent mean ± SEM of at least 3 independent experiments with intra-experiment duplicates. The white bar (-) represents absence of PS; the black bar (+) represents presence of PS.

In order to maintain viability of bacteria when topically exposed to RHG, two essential technical steps were investigated. These were a) the impact of topically applied agar which can be used as a vehicle to keep the bacteria localized on the surface of RHG thus preventing bacteria from spilling over into the culture medium *via* the edges of the transwell, and b) the impact of the absence of PS on RHG which would be necessary to maintain viable bacteria for 3 days. Clearly the topical presence of agar and the absence of PS in the culture medium did not influence RHG histology (**Figure 1**). In all experimental conditions, a well stratified and differentiated epithelium was present resembling native gingiva in line with our previous publications (Buskermolen et al., 2016). The proliferation marker Ki67 and differentiation marker keratin10 expression remained unaltered, with Ki67 positive cells located in the basal layer and keratin10 expression in suprabasal cells (**Figure 1A**). No significant difference in the Ki67 proliferation index was found between the four experimental conditions (unexposed group in absence of PS: 26.1 ± 5.81%; unexposed groups in presence of PS: 19.4 ± 5.96%; agar group in absence of PS: 20.7 ± 4.78%; agar group in absence of PS 21.1 ± 8.40%).

Furthermore, metabolic activity, which correlates to cell viability remained unaltered by the presence of agar or the absence of PS (**Figure S1**). In order to determine whether the presence of agar and absence of PS influenced the innate immune response, secretion of (pro-) inflammatory cytokines and chemokines IL-6, IL-8, CCL2, CCL5, CCL20 and CXCL1 was determined in RHG culture supernatants. No significant differences were observed (**Figure S1**). In order to confirm that the method of applying bacteria within agar to the surface of RHG prevented spilling over into the culture medium, 100µL culture media from bacteria exposure groups (absence of PS) were collected at media refreshment daily, plated on BHI or BA agar and incubated anaerobically for 3 days. No colonies could be observed in any sample.

### Co-culture of RHG with commensal *S. gordonii* and pathogenic *A. actinomycetemcomitans* does not influence RHG viability or histology

It was next determined whether bacteria cultured within agar applied topically to RHG would influence RHG. Compared to control cultures which were only exposed to agar, co-culture with *S. gordonii* or *A. actinomycetemcomitans* did not influence RHG proliferation or differentiation since a well stratified and differentiated epithelium could be observed in all experimental conditions (**Figure 1B**). The viability of RHG was unaltered after bacteria exposure as assessed by Ki67 which stains actively dividing basal layer keratinocytes which form the stratified epithelium. No significant differences in Ki67 index could be observed between the control and bacteria exposed groups (data not shown). Furthermore, with the exception of the high density of *A. actinomycetemcomitans* which resulted in a slightly higher metabolic activity when cultured in the presence of PS compared to RHG without PS, metabolic activity of the co-culture conditions was similar to control agar exposed RHG (**Figure 1C**).

### Bacteria remain viable when topically applied to RHG for up to 3 days

**Figure 2:**
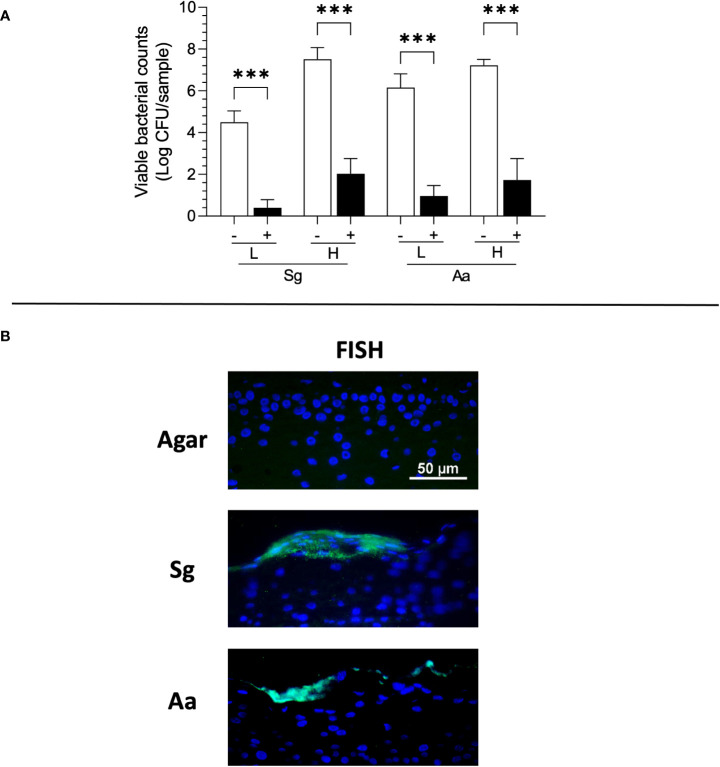
Viability and Fluorescence *in situ* Hybridization (FISH) staining of *S. gordonii (Sg*) and *A. actinomycetemcomitans* (Aa) 3 days after topical application to RHG. **(A)** The bacterial viability is presented as log CFU/sample (bacteria inoculation density: L =low (10^6^ CFU/sample), H = high (10^8^ CFU/sample)). One-way ANOVA followed by Fisher’s LSD test was performed to detect the influence of PS on bacterial viability. ***p<0.001. The white bar (-) represents absence of PS; the black bar (+) represents presence of PS. **(B)** Bacteria (green) are detected using FISH on the surface of epithelium. Keratinocyte nuclei are counterstained with DAPI (blue).

Previously, we have shown that the topically applied bacteria in Hanks’ balanced salt solution HBSS to RHG in the presence of PS rapidly decreased viability within 1 day (Shang et al., 2018). Therefore, we next determined whether bacteria would remain viable if cultured within agar applied topically to RHG in the absence of PS. Independent of the density applied (high or low), both bacterial species remained viable and were 4-5 fold more abundant at log scale than in culture conditions where PS was present (**Figure 2A**). In the absence of PS, CFU of both the commensal *S. gordonii* and pathogenic *A. actinomycetemcomitans* were similar after 3 days exposure to the initial inocula, the CFU of which were 7.9 ± 0.1 and 8.1 ± 0.1 log CFU/sample for *S. gordonii* (high) and *A. actinomycetemcomitans* (high) respectively. We further performed FISH to visualize the location of the bacterial cells (**Figure 2B**). Both bacteria species were located on the surface of the gingiva epithelium and appeared to be trapped within the agar matrix. The bacterial cells were not observed within the epithelium. Moreover, the bacterial cells were not observed in any RHG culture medium samples obtained from beneath the transwell when co-incubated with bacteria.

### Differential cytokine release after co-culture of RHG with commensal *S. gordonii* or pathogenic *A. actinomycetemcomitans*

**Figure 3:**
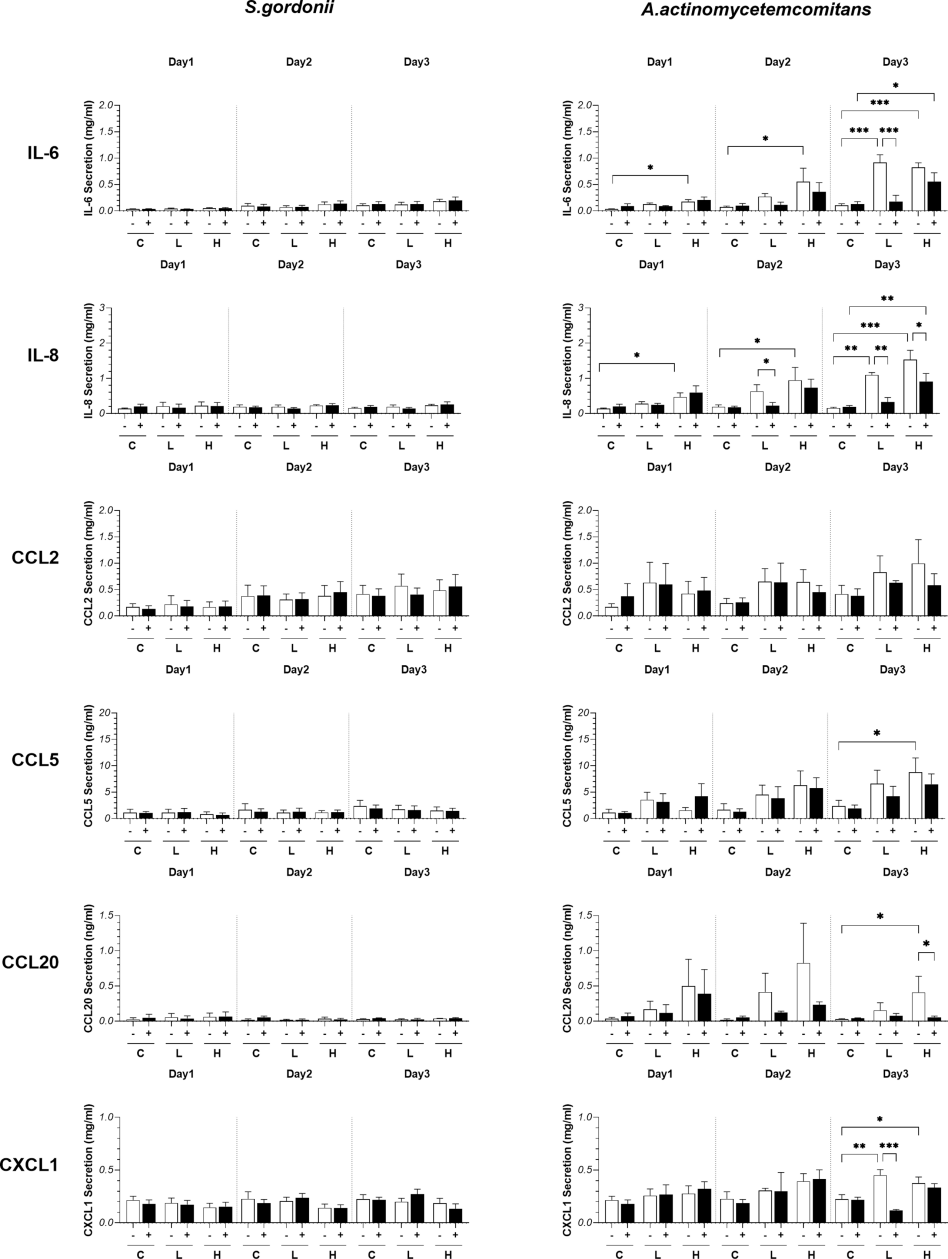
Influence of *S. gordonii (*Sg) and *A. actinomycetemcomitans* (Aa) on cytokine release. (pro-)inflammatory cytokine and chemokine secretion after bacteria exposure (day 1: 0 - 24h; day 2: 24 - 48h; day 3: 48 - 72h). Two-way ANOVA followed by Fisher’s LSD test was performed to determine the influences of PS and bacteria density on cytokines release. *p<0.05, **p<0.01, ***p<0.001. Data represent mean ± SEM of at least 3 independent experiments with intra-experiment duplicates. The white bar (-) represents absence of PS; the black bar (+) represents presence of PS. C: agar vehicle control; L: low bacterial inoculation density; H: high bacterial inoculation density.

In order to determine whether the presence of bacteria influenced secretion of (pro-) inflammatory cytokines and chemokines, RHG culture supernatants were assessed by ELISA. Co-culture of RHG with *S. gordonii*, in the presence or absence of PS, did not influence IL-6, IL-8, CCL2, CCL5, CCL20 or CXCL1 secretion. Cytokine secretion remained low and comparable to agar exposed RHG over the 3 days exposure period (**Figure 3**). However, when RHG were co-cultured with *A. actinomycetemcomitans*, (with the exception of CCL2 which remained comparable to agar controls) all cytokine levels increased when PS was absent (**Figure 3**). This was particularly visible in the day 2 and day 3 samples which contain cytokines secreted within the 24 hours preceding the collection time point, with increased cytokine levels reaching 10-fold higher compared to controls for IL-6 and IL-8. In general, a lower but still significant cytokine secretion was observed when RHG were cultured in the presence of PS and this was particularly apparent for IL-6, IL-8 and CXCL1 secretion.

### Comparison of live and dead *A. actinomycetemcomitans* on cytokine secretion

**Figure 4:**
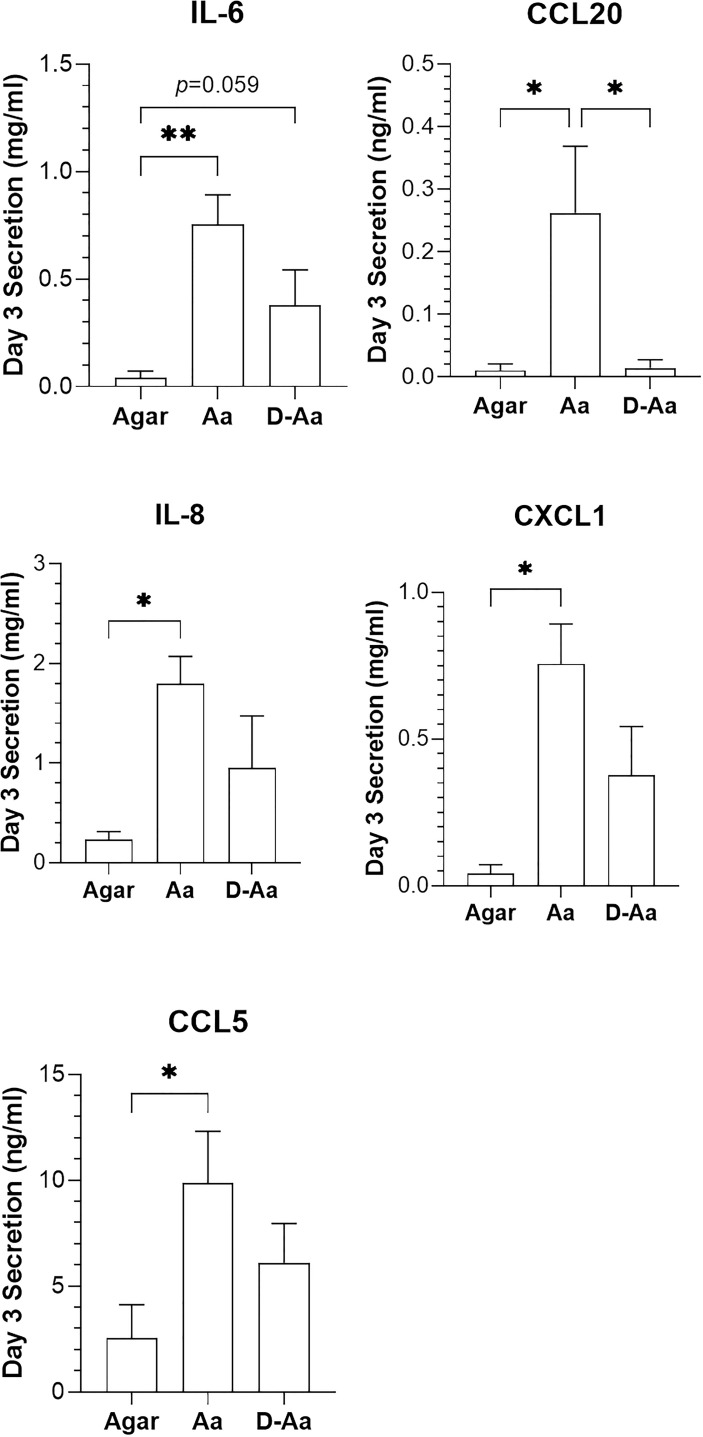
Comparison of cytokine release 3 days after RHG was exposed to live or dead *A.actinomycetemcomitans* (absence of PS). Live *A. actinomycetemcomitans (*Aa) and heat-killed *A. actinomycetemcomitans (*D-Aa) were applied to RHG at a density of 10^8^ CFU/sample. One-way ANOVA followed by Fisher’s LSD test was performed to detect the differences among agar, Aa and D-Aa groups. *p<0.05, **p<0.01. Data represent mean ± SEM of at least 3 independent experiments each with an intra-experiment duplicate.

To further investigate the added value of viable bacteria in host – microbe immune responses, cytokines secreted by RHG after exposure to high density living or 100% heat-killed *A. actinomycetemcomitans* (equivalent to 8.3 ± 0.1 log CFU/sample living cells) were next compared (**Figure 4**). Notably, whereas living bacteria co-cultured in the absence of PS resulted in a significant increase in IL-6, IL-8, CCL5, CCL20 and CXCL1 secretion on day 3, the heat-killed *A. actinomycetemcomitans* resulted in much lower increase in secretion of these cytokines.

### Bacteria increase antimicrobial peptides

**Figure 5:**
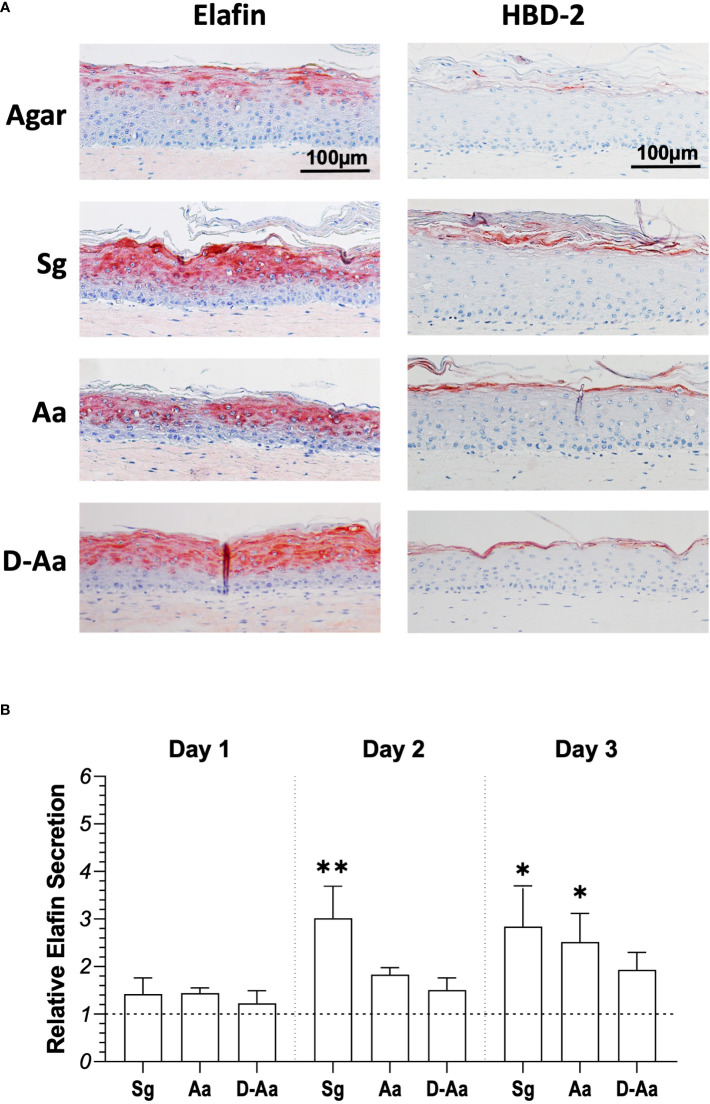
Antimicrobial peptide (AMPs) expression in RHG epithelia after exposure to living or heat-killed bacteria for 3 days. **(A)** Immunohistochemical staining (Elafin/HBD-2) of RHG after 3 days bacteria exposure (bacteria inoculation density: 10^8^ CFU/sample, absence of PS). **(B)** Elafin secretion in RHG culture medium was assessed by ELISA for 3 days (day 1: 0 - 24h; day 2: 24 - 48h; day 3: 48 - 72h). Results are expressed relative to agar vehicle control. Kuskal-Wallis followed by Dunn’s multiple comparison test was performed to detect the differences between bacteria exposure and agar control groups. *p<0.05, **p<0.01. Data represent mean ± SEM of at least 3 independent experiments with intra-experiment duplicates. Sg: live *S. gordonii*; Aa: live *A. actinomycetemcomitans.* D-Aa: heat-killed *A. actinomycetemcomitans*. The dash line represents agar vehicle control.

The observed differential cytokine secretion after commensal *S. gordonii* or pathogenic *A. actinomycetemcomitans* exposure next prompted the investigation of antimicrobial peptide expression in RHG co-cultured with bacteria. Elafin (also named skin-derived antileukoprotease (SKALP)) and HBD-2 are antimicrobial peptides (AMPs) which are expressed at low levels in the upper epithelium layer of RHG (**Figure 5**). When RHG were co-cultured with live *S. gordonii*, live or dead *A. actinomycetemcomitans (*in the absence of PS) for 3 days an increased expression of both proteins was observed, in particular for Elafin (**Figure 5A**). To further quantify the secretion of Elafin, culture supernatants were analyzed by ELISA (**Figure 5B**). RHG exposed to living *S. gordonii* showed a 3-fold increase in Elafin secretion compared to agar control in both day 2 and day 3 culture supernatants. In contrast, a significant increase in Elafin secretion after live *A. actinomycetemcomitans* exposure was only observed at day 3.

## Discussion

Here we describe a technical advance which enables co-culture of RHG together with living commensal *S. gordonii* or pathogenic *A. actinomycetemcomitans* bacteria for a duration of 3 days. This development enables investigation of dynamic human host–microbe interactions over an extended period *in vitro*. Literature reviews describing work from other groups show that the few studies performed mainly report RHG exposed to a single- or dual-species of oral bacteria for maximally 48 hours (Dongari-Bagtzoglou and Kashleva, 2006; Tabatabaei et al., 2020). Most of these reports described a disrupted tissue integrity, decreased viability and increase in apoptosis of host tissues within 48 hours (Andrian et al., 2004; Gursoy et al., 2010; de Carvalho Dias et al., 2018; Morse et al., 2018; Beklen et al., 2019). One report described a model where a multi-species biofilm was co-incubated with RHG for 3 days without affecting the viability of RHG (De Ryck et al., 2014). However, in that model, the biofilms were cultured on a transwell membrane and not in direct contact with the RHG. In contrast, our model describes for the first time, a direct contact interaction between host and bacteria, which is similar to *in vivo*. We demonstrated that the viabilities of both host and bacteria, and tissue integrity could be preserved for the entire 3-day culture period.

Our technical advance was achieved by introducing 2 key changes into our previously reported studies. The first being that the bacteria were applied topically to RHG in a soft, low concentration of agar containing sufficient nutrients to maintain viability or promote growth of bacterial cells and which also solidified thus keeping the bacteria localized on the surface of RHG. In one of our previous studies, bacteria were applied in suspension which could spill over into the RHG culture medium *via* the edges of the transwell and therefore PS was required to prevent contamination so that a prolonged co-culture period of 7 days was reached. However, this resulted in a rapid decrease to 10% of bacterial viability within 24 hours and eventually death of all bacteria soon afterwards (Shang et al., 2018). This motivated our second protocol adaption which was to omit PS from the RHG culture medium from the time that bacteria were topically applied. We expected that this would permit optimal conditions for stable culture of bacteria on RHG within the soft agar gel. Indeed, our results showed when RHG-bacteria co-cultures were grown in the presence of PS, the viable counts decreased considerably but when grown without PS, the bacterial viability were unaffected. This finding also indicated that the PS added to the cell culture medium can diffuse through the RHG into the agar containing the bacteria and reduced bacterial viability, which inevitably affects the studied host-microbe interactions.

It has been described by others that PS, when added throughout the culture period, has a detrimental effect on keratinocytes. In particular, these antibiotics suppressed keratinocyte proliferation and differentiation in monolayer culture as well as the formation of a multilayered reconstructed epidermis (Neftel and Hübscher, 1987; Llobet et al., 2015; Nygaard et al., 2015). In our study, we did not observe any differences in RHG viability, proliferation, differentiation or inflammatory cytokine release when cultured in the presence or absence of PS or when agar was present. However it should be noted that, in contrast to these studies which only had an epidermis, our RHG also has a fibroblast populated hydrogel compartment which will substantially support growth of the epithelium (El-Ghalbzouri et al., 2002).

Having established a stable model for investigating host-microbe interactions, we next went on to show that commensal *S. gordonii* and pathogenic *A. actinomycetemcomitans* exerted differential responses in RHG. Whilst neither bacterium significantly influenced RHG viability or tissue histology, differential effects on (pro-)inflammatory cytokine and chemokine secretion and expression of AMPs were observed. *S. gordonii* exposure resulted in a negligible inflammatory response whereas *A. actinomycetemcomitans* strongly stimulated cytokine and chemokine secretion indicating that the later had initiated TLR signaling and activation of the inflammazone, which, *in vivo*, would be expected to combat invasion of the pathogen into the host tissues in order to maintain homeostasis (Wells et al., 2011; McClure and Massari, 2014; Shang et al., 2019). Previous studies have shown that virulence factors, such as lipopolysaccharide or genomic DNA of *A. actinomycetemcomitans* were able to induce proinflammatory cytokine production in human gingival fibroblasts. These factors were *A. actinomycetemcomitans* specific, which might explain the RHG differential expression observed between *A. actinomycetemcomitans* and *S. gordonii* (Patil et al., 2006; Soto-Barreras et al., 2017)

So far, only one study has examined the differential inflammatory responses of gingival epithelial cells to 24 hours exposure of *S. gordonii* and *A. actinomycetemcomitans* (Dickinson et al., 2011). This study showed that both bacteria induced IL-6 and IL-8 secretion, but the levels of induction by *A. actinomycetemcomitans* were lower than *S. gordonii*. The different IL-6 and IL-8 secretion patterns in Dickinson et al. and our study may be due to the different host model used: the previous study used multi-layer epithelial cells whereas this study used a 3D organotypic model consisting of epithelial and fibroblast cells (Dickinson et al., 2011; Shang et al., 2018). Moreover, the bacterial strains used in these two studies might also play a role. The differential host responses to commensals and pathogens have been the focus of several studies. However, the findings were inconsistent. Ingendoh-Tsakmakidis and Shang showed pathogenic biofilms induced lower cytokine secretion of RHG, as compared to commensal biofilms, whereas Brown found the opposite trend: the “health-associated” *Streptococcus* biofilms had little inflammatory effect on the RHG tissue but the “gingivitis-associated” biofilms induced significant inflammatory response, which is in line with the result of current study (Shang et al., 2019; Ingendoh-Tsakmakidis et al., 2019; Brown et al., 2019). Taken together, the contrasting results are likely due to different RHG models and different types of biofilms, single- or multi-species, employed in the studies.

Moreover, the expression of AMPs (Elafin and HBD-2) increased in the upper regions of RHG epithelium after exposure to both bacteria, in line with our previous studies (Buskermolen et al., 2018; Shang et al., 2018). However, substantially more Elafin secretion was observed after commensal *S. gordonii* exposure compared to the pathogenic *A. actinomycetemcomitans*. This finding is supported by another report, where a periodontal pathogen *Porphyromonas gingivalis* significantly reduced Elafin gene expression and secretion in gingival keratinocytes (Yin et al., 2010). This would indicate that the commensal bacteria can initiate a more protective response from RHG than the pathogenic bacteria. Importantly, our results also show that the observed inflammatory response from living *A. actinomycetemcomitans* (without PS) is more than just a question of TLR activation by bacterial cell membrane components (e.g., Lipopolysaccharides) since when the same number of heat killed bacteria were applied to RHG the inflammatory cytokine response was significantly lower.

As with all complex *in vitro* models our host-microbe model does have limitations and there is still room for further improvement. In this study, the RHG was constructed using TERT immortalized gingiva keratinocyte and fibroblast cell lines. In the past the cell line model was extensively compared with the RHG constructed from primary cells and was found to show excellent correlation with regards to histology, biomarker expression and cytokine secretion (Buskermolen et al., 2016). Since the use of primary cells is accompanied with extensive limitations such as availability and logistics of receiving fresh tissue biopsies, small biopsies resulting in limited scalability (low number of replicate RHG within an experiment), and high risk of infection from oral microbiota, it was decided to use the TERT RHG model in this study. However, this is accompanied by the fact that our results represent a single donor for the keratinocytes and a non-matched single donor for the fibroblasts and therefore donor variation is not possible to study in the current TERT cell line model. Notably, immune cells are absent from the current host-microbe interaction model. Since antigen presenting cells, such as Langerhans cells, play an important role in the host’s response to microbes, these immune cells will now be incorporated into the model. Already we have been able to show that MUTZ-3 derived Langerhans Cells incorporated into RHG maintain their functionality and plasticity as shown by their ability to mature and migrate from the epithelium to the fibroblast hydrogel upon exposure to contact sensitizers (Kosten et al., 2016).

In this study, we used a single species for the microbial challenge which is less complex than the *in vivo* microbiome, and therefore the RHG response resembles a conditioned host reaction. Furthermore, the amount of *S. gordonii* and *A. actinomycetemcomitans* applied for practical reasons needs to be carefully interpreted as *in vivo* these bacteria constitute part of the complex oral microbiome with differential proportions which also vary among oral sites (Mark Welch et al., 2019). For future studies, it would be most interesting to expose RHG to a mixture of *S. gordonii* and *A. actinomycetemcomitans*, as enhanced virulence has been reported for this mixture.

In conclusion, we describe here a novel model to investigate human host-microbiome interactions which is stable for at least 3 days in aerobic culture conditions. Such a model has potential to investigate multi-species biofilms and the influence of antibiotics and probiotics in the future and importantly to replace current animal models which do not adequately represent human host-microbe interactions.

## Data availability statement

The raw data supporting the conclusions of this article will be made available by the authors, without undue reservation.

## Ethics statement

This study did not involve any living humans or animals, neither did it involve biological samples derived from humans and therefore no ethical consent was required for this study. It only involved immortalized human cell lines and bacterial strains which were obtained as described in Methods.

## Author contributions

YZ, methodology, investigation, formal analysis, data curation, and writing – original draft. LS, methodology, investigation, formal analysis, and writing – original draft. SR, methodology and investigation. BPK, writing – review and editing. SG, conceptualization, investigation, writing – original draft, writing – review and editing, supervision. DD, conceptualization, methodology, investigation, writing – original draft, writing – review and editing, and supervision. All authors gave their final approval and agreed to be accountable for all aspects of the work.

## Funding

YZ is supported by China Scholarship Council (Number: 201808440350) for the research.

## Conflict of interest

The authors declare that the research was conducted in the absence of any commercial or financial relationships that could be construed as a potential conflict of interest.

## Publisher’s note

All claims expressed in this article are solely those of the authors and do not necessarily represent those of their affiliated organizations, or those of the publisher, the editors and the reviewers. Any product that may be evaluated in this article, or claim that may be made by its manufacturer, is not guaranteed or endorsed by the publisher.
