# Supplementary figures and images for: Stable reconstructed human gingiva–microbe interaction model: Differential response to commensals and pathogens
Source: Front Cell Infect Microbiol. 2022 Oct 20;12:991128. doi: 10.3389/fcimb.2022.991128 (PMC9631029; doi:10.3389/fcimb.2022.991128)

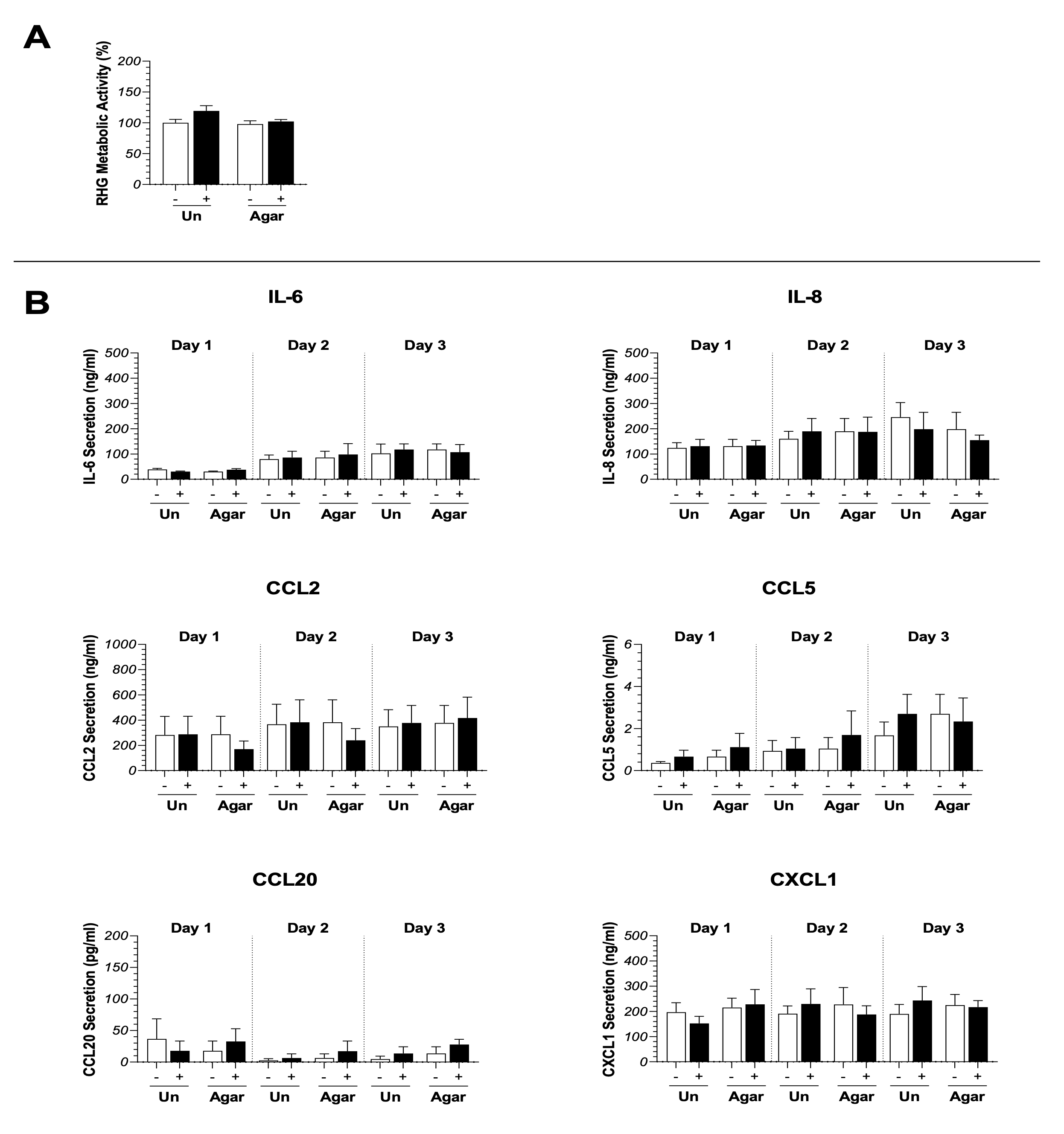

Supplement: Supplementary file 1 [file Image_1.jpg]
